# Correction to “ApoE expression in macrophages communicates immunometabolic signaling that controls hyperlipidemia‐driven hematopoiesis & inflammation via extracellular vesicles”

**DOI:** 10.1002/jev2.12375

**Published:** 2023-10-26

Phu, T. A., Ng, M., Vu, N. K., Gao, A. S., & Raffai, R. L. (2023). ApoE expression in macrophages communicates immunometabolic signaling that controls hyperlipidemia‐driven hematopoiesis & inflammation via extracellular vesicles. Journal of Extracellular Vesicles, 12, e12345. https://doi.org/10.1002/jev2.12345

In the originally published article, the incorrect GSE accession number was reported in Section 4.8. The incorrect number, GSE237727, should instead be GSE237730.

We apologize for this error.

